# How rice adapts to high temperatures

**DOI:** 10.3389/fpls.2023.1137923

**Published:** 2023-03-17

**Authors:** Huimin Ren, Jingpei Bao, Zhenxian Gao, Daye Sun, Shuzhi Zheng, Jiaoteng Bai

**Affiliations:** ^1^ Ministry of Education Key Laboratory of Molecular and Cellular Biology, Hebei Research Center of the Basic Discipline of Cell Biology, Hebei Collaboration Innovation Center for Cell Signaling and Environmental Adaptation, Hebei Key Laboratory of Molecular and Cellular Biology, College of Life Sciences, Hebei Normal University, Shijiazhuang, China; ^2^ Shijiazhuang Academy of Agriculture and Forestry Sciences, Wheat Research Center, Shijiazhuang, China

**Keywords:** rice, high temperature, heat stress, survival strategies, sustainable agriculture, adaptive traits phenotyping

## Abstract

High-temperature stress affects crop yields worldwide. Identifying thermotolerant crop varieties and understanding the basis for this thermotolerance would have important implications for agriculture, especially in the face of climate change. Rice (*Oryza sativa*) varieties have evolved protective strategies to acclimate to high temperature, with different thermotolerance levels. In this review, we examine the morphological and molecular effects of heat on rice in different growth stages and plant organs, including roots, stems, leaves and flowers. We also explore the molecular and morphological differences among thermotolerant rice lines. In addition, some strategies are proposed to screen new rice varieties for thermotolerance, which will contribute to the improvement of rice for agricultural production in the future.

## 1 Background

High-temperature stress is an important environmental factor affecting crop growth and yield (Yang et al., 2017). In 2017, four independent studies estimated that for every 1°C increase in the average global temperature, rice (*Oryza sativa*) yields would decrease by 3.2%, maize (*Zea mays*) yields by 7.4%, wheat (*Triticum aestivum*) yields by 6%, and soybean (*Glycine max*) yields by 3.1% (Zhao et al., 2017). Therefore, mitigating the decreases in yield caused by high temperatures by developing and cultivating new thermotolerant crop varieties has emerged as an important strategy for sustainable agriculture. Rice represents the main food source for nearly half of the world’s population, accounting for 21% of total global caloric intake. The cultivation area of rice accounts for 11% of the total cultivated land area worldwide. It is estimated that global rice consumption will increase from 480 million tons in 2014 to nearly 550 million tons in 2030 (Yuan et al., 2021). Rice yield is estimated to decrease by up to 10% with every 1°C increase in land surface temperature (Peng et al., 2004). Given the frequency of extreme heat events, it is important to identify existing thermotolerant rice varieties and develop new ones.

In this review, we explore the effects of high temperature on rice at different stages of growth. We then explore the effects of high-temperature stress on cellular processes and phytohormones in rice. Finally, we examine the mechanisms by which thermotolerant rice varieties acclimate to high temperature. Understanding these effects and mechanisms will provide a foundation for producing new thermotolerant rice varieties, which will be essential for the future of agriculture.

## 2 Effects of high temperature on rice at different stages

**Figure 1:**
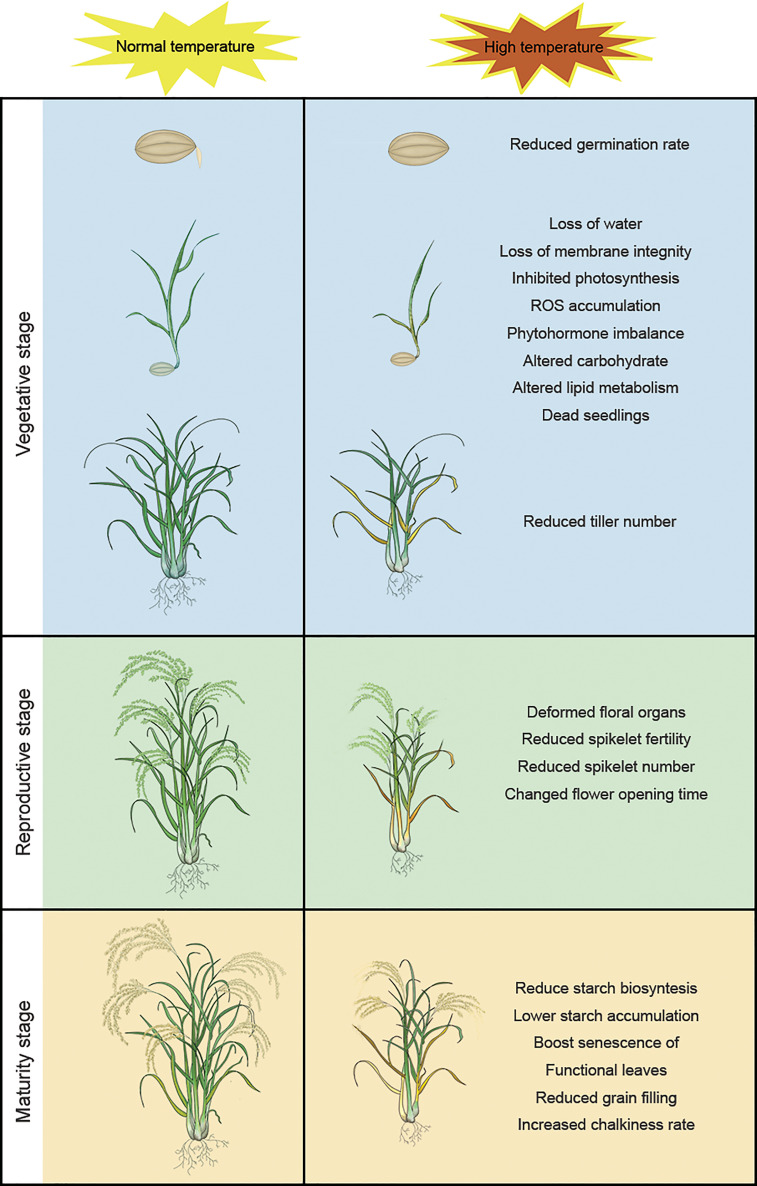
High temperature has different effects on rice at different stages of the plant life cycle: reduced germination rate, loss of water, loss of membrane integrity, inhibited photosynthesis, ROS accumulation, phytohormone imbalance, altered carbohydrate, altered lipid metabolism, dead seedlings, and reduced tiller number at the vegetative stage; deformed floral organs, reduced spikelet fertility, reduced spikelet number, changed flower opening time, reduced spikelet fertility, reduced spikelet number, and altered flower opening time at the reproductive stage; reduce starch biosynthesis, lower starch accumulation, boost senescence of functional leaves, reduced grain filling and increased chalkiness rate at the maturity stage.

The life cycle of rice is divided into vegetative (germination, seedling growth, and tillering) and reproductive (booting, heading, flowering, and maturation/grain filling) stages; high temperatures have different effects on plants at different stages of development (Zhang C et al., 2018). Rice plants are particularly sensitive to temperature stress during reproductive growth and maturation, when exposure to high-temperature stress can significantly affect development and yield (Lawas et al., 2019; Chen et al., 2020). The two subspecies, *Japonica* and *Indica*, have different domestication origins. *Japonica* and *Indica* originated in temperate regions and tropical regions, respectively. *Indica* is more thermotolerant than *Japonica* and has different morphological and physiological characteristics (Lee et al., 2002; Lee et al., 2017). So, these geographic origins should be taken into account, when different kinds of species were used for high temperature treatment. Therefore, during the domestication of modern rice varieties, the two subspecies were used as backgrounds of their respective varieties. **Figure 1** shows the impact of high temperature on different developmental stages of rice.

### 2.1 Influence of high temperature on vegetative growth

Vegetative growth includes seed germination, early seedling growth, and tillering. Long-term exposure to high temperatures affects the germination rate of rice seeds (Liu et al., 2015). In seedlings, high temperature can destroy cell membranes, inhibit photosynthesis, and increase oxidative damage, leading to increased water loss, wilting, damage to root growth, and even plant death (Bahuguna et al., 2015; Liu et al., 2018). To mitigate these effects, rooting agents such as iron chelators, calcium, silicone, and gibberellins can be added during sowing to improve the seed germination rate, promote root growth, and reduce the effects of high temperatures on the resulting plants (Tiwari et al., 2022).

Tillering determines the structure and yield of rice plants and is thus an important agronomic trait of rice. Tillers develop from the leaf axillary bud on the mother stem and grow at an angle centered on the main stem (Wang et al., 2018; Luo et al., 2021). Tiller number and angle affect yield: tillers growing at a large angle reduce harvest efficiency, planting density, and yield, whereas tillers growing at a small angle increase disease risk (Dias de Oliveira et al., 2015; Hu et al., 2020). High temperature can decrease tillering and thus panicle number in rice. For example, Soda reported that panicle number and yield per plant decreased by 35% and 28%, respectively, in rice plants subjected to high-temperature stress (Soda et al., 2018).

Studies aimed at mitigating these effects have focused on the regulation of tillering. For example, heterologous overexpression of *TaGAMYB1* (encoding a GAMYB-like family transcription factor) or overexpression of *FIBRILLIN 1* (*OsFBN1*) or *OsSTVB-I* (a rice stripe virus resistance gene) improved thermotolerance and increased tiller number in rice (Li et al., 2019; Hayano-Saito and Hayashi, 2020; Wang et al., 2012). In addition, the tiller angle between the main stem of rice and its side tillers also directly affects the photosynthetic rate, and thus affects the rice yield per unit area (Li et al., 2020a). Recent studies have shown that the expression of *HEAT SHOCK FACTOR A2d* (*HSFA2d*) increases rapidly only 15 min after HS, and the formation of rice tiller angle can be regulated by *HSFA2D-LA1* (*LAZY1*) pathway in rice (Zhang N et al., 2018; Hu et al., 2020; Prerostova et al., 2021). Therefore, the phenotypic characteristics of tillers can also be used as one of the screening criteria for thermoresistant varieties.

### 2.2 Influence of high temperature on reproductive development

The reproductive stage includes booting, heading, and flowering. We will discuss maturation, which includes grain filling, in Section 2.3. The reproductive period, especially heading and flowering, is very sensitive to high-temperature stress (Matsui et al., 2015). Rice belongs to the Poaceae family. The spikelet, representing the basic structure of the Poaceae inflorescence, is composed of stamens, pistils, glumes, and lemmas (Ren et al., 2020). High temperature during the reproductive period primarily affects the fertility and number of spikelets, resulting in a serious decline in rice yield. As early as 1978, Satake and Yoshida reported that exposing rice to temperatures above 35°C for about five days during the reproductive period resulted in spikelet sterility and failure to produce seeds (Satake and Yoshida, 1978). The effect of high temperature on the reproductive period was greater before than after flowering. Exposing pre-flowering spikelets to 33.7°C for one hour was sufficient to cause sterility, but exposing post-flowering spikelets to 38°C or 41°C for one hour did not cause sterility (Jagadish et al., 2007; Ishimaru et al., 2010; Shi et al., 2018).

Stamens are particularly sensitive to high temperature. Indeed, Miura et al. reported that pistils could be successfully fertilized by hand pollination even after five-day exposure to 41°C (Miura et al., 2011). High temperature has several negative effects on rice stamens. Firstly, high temperature interferes with meiosis during pollen development, resulting in the formation of sterile pollen (Endo et al., 2009). Secondly, high temperature inhibits pollen dehiscence and decreases the swelling ability of pollen grains, thus reducing the amount of pollen falling on a stigma and affecting the fertilization rate (Arshad et al., 2017; Hu et al., 2021). Thirdly, the moisture content of pollen grains is crucial to both the formation and diffusion of pollen grains. When pollen falls onto a stigma, the moisture content changes according to environmental conditions; high temperature plays a role in disrupting this process (Das et al., 2014; Shrestha et al., 2022). Finally, high temperature strongly reduces the amount of protein present in pollen, thereby reducing the germination ability of pollen and the elongation rate of the pollen tube, leading to spikelet sterility (Arshad et al., 2017; Jagadish, 2020; Shrestha et al., 2022).

Some thermotolerant rice varieties rapidly release pollen early during flowering, but many thermosensitive varieties fail to release pollen in time to effect fertilization. For example, in the thermotolerant rice variety Nagina 22 (N22), pollen dehiscence occurs rapidly upon opening of the glumes, and the released pollen settles on the stigma (Kobayashi et al., 2015). The anther cavity of thermotolerant *Japonica* rice varieties (Akitakomachi, Nipponbare) are well developed, and the septum breaks rapidly when the pollen expands, resulting in high spikelet fertility (Matsui and Omasa, 2002). High temperature also causes decreases in pollen protein content and viability. Indeed, a typical characteristic of a thermotolerant variety is its strong pollen viability (Zhao et al., 2018). Pollen tubes are also extremely sensitive to high temperature, which affects grain yield. Therefore, studying the morphology and physiology of floral organs from different thermosensitive and thermotolerant varieties may help breeders screen varieties for thermotolerance (Matsui and Omasa, 2002).

Approaches to mitigating the effect of high temperature on reproductive development have focused on identifying the underlying genetic factors. For example, Ye et al. identified a QTL (*qHTSF4.1*) on chromosome 4 in 24 rice varieties. Compared with rice varieties without *qHTSF4.1*, most varieties with this QTL showed increased spikelet fertility under high-temperature stress during the reproductive period, thus improving yield (Ye et al., 2015). Therefore, in addition to detecting pollen viability, changes in the expression levels of related target genes can also be detected after high-temperature stress to evaluate the changes in pollen metabolism and phytohormone levels, representing another effective method for identifying thermotolerant rice varieties.

In addition, rice varieties have different flowering duration and significantly different flower opening time (FOT), ranging from early morning to midnight. High temperatures shorten the flowering time, limiting the time for pollination (Jagadish et al., 2007; Djanaguiraman et al., 2020). Therefore, changing the FOT can help protect rice from high-temperature stress, thereby reducing damage during flowering. This early-morning flowering (EMF) strategy has been effectively applied in rice. This strategy is to breed cultivars that escape high temperature at flowering because of their EMF trait has been effectively applied in rice.

Under simulated continuous warming, Nanjing 11 began flowering after 7 am, with one flowering peak between 9 and 10 am and one between 11 and 12 am. However, *qEMF3* advanced the flowering peak of Nanjing 11 to 7-8 am, thereby improving thermotolerance (Hirabayashi et al., 2015; Jagadish, 2020). In addition, when post-flowering stage is exposed to high night temperatures, respiration is accelerated, resulting in higher carbon loss, and affecting starch accumulation, thus reducing rice yield (Bahuguna et al., 2017; Impa et al., 2021). In conclusion, early flowering can protect spike heads and preserve fertility when plants experience high temperatures. We can systematically evaluate the start and peak of the FOT, select suitable varieties, or modify the FOT to protect crops from high temperatures during propagation and thus improve yields.

### 2.3 Influence of high temperature on maturation

Grain filling occurs during the maturity stage and involves the conversion of sucrose produced by photosynthesis into starch, which is the major carbohydrate in rice and an important determinant of grain quality and yield (Tang et al., 2018). The starch in rice grains is mainly derived from sugars produced in the grains after anthesis and from sugars that are redistributed from other vegetative tissues, such as stems and leaf sheaths. For reactivating assimilates stored in vegetative tissues and transferring them to grains, whole-plant senescence should be initiated (Yang and Zhang, 2006). Delaying the senescence results in insufficient grain filling, with large amounts of carbohydrates remaining unused in the straw (Plaut et al., 2004).

High temperature during grain filling can reduce rice yields by 50% (Sreenivasulu et al., 2015). It can also cause the failure of grain filling in rice and wheat by affecting the accumulation of starch granules, ultimately resulting in yield losses (Chen et al., 2017; Impa et al., 2021). High-temperature stress during grain filling also induces DNA methylation in the promoters of abscisic acid (ABA) catabolic genes and α-amylase genes, which can delay the germination of the resulting seeds (Suriyasak et al., 2020).

High temperature affects starch accumulation in rice grains *via* the following mechanisms: Firstly, high temperature can shorten the grain filling period, resulting in insufficient grain filling and inadequate starch accumulation, thus reducing grain yield (Chen et al., 2021b). Secondly, high temperature can reduce the gene expression and bioactivity of key enzymes involved in the conversion of sucrose to starch in endosperm, reducing the rate of starch synthesis and thus affecting total starch content and starch accumulation patterns, especially the amylose content of endosperm starch (Yamakawa and Hakata, 2010; Zhang et al., 2021; Shirdelmoghanloo et al., 2022). Thirdly, high temperature can inhibit photosynthesis in other vegetative organs such as stems and leaf sheaths, resulting in an insufficient supply of fixed carbon from vegetative organs to spikelets, a slower grain filling rate, and a lower grain weight (Yang and Zhang, 2010).

Spermidine (Spd) is an important bioactive polyamine in plants. Exogenous Spd treatment enhances thermotolerance in seeds by regulating starch and polyamine metabolism and reducing high-temperature-induced damage during grain filling. Spd treatment increased the accumulation of OsSAP5 (stress-associated protein 5, an A20/AN1 zinc finger domain protein). When *OsSAP5* was heterologously overexpressed in *Arabidopsis thaliana*, the 1,000-grain weight and thermotolerance of seeds were significantly enhanced. OsSAP5 may be involved in Spd-mediated thermotolerance during seed development (Chen et al., 2021b). Fibrillin 1 (OsFBN1) and the heterodimers formed by ONAC127/129 (two seed-specific NAM/ATAF/CUC domain transcription factors) are also involved in plant growth and grain filling under high-temperature stress (Li et al., 2019; Ren et al., 2021). Rice mutants with deletions of *CPHSP70-2* are sensitive to high temperatures and show substantial chalkiness in grains, which seriously affects their yield and quality (Jiang et al., 2021; Yang et al., 2021).

Some studies have shown that grain filling is related to the overall thermotolerance of plants, and most thermosensitive lines show impaired grain filling under high-temperature stress (He et al., 2018; He et al., 2021). The loss-of-function mutants of *ERECTA* are thermosensitive, and their seeds are smaller than those of the wild type (WT) (Shen et al., 2015; Wu et al., 2022b). High temperature not only reduced the size and yield of rice seeds, but also affected the quality of endosperm (Nevame et al., 2018). The grains of rice grown under normal conditions are round, full, and transparent after filling. However, the rice chalkiness rate (chalkiness is the opaque part of the endosperm in the rice kernel and contain a lower density of starch granules as compared to vitreous ones) increased significantly and the grain weight decreased after high-temperature stress (Wada et al., 2019; Wang et al., 2022a). This has seriously affected the quality of rice and the cooking taste of consumers. Therefore, thermotolerant varieties can be screened by measuring seed size, weight, and the frequency of chalky grains.

## 3 Heat stress responses in rice

How do plants sense high temperatures? Numerous studies on the model plant *Arabidopsis* have revealed multiple “thermosensors,” including the Ca^2+^ channel proteins CNGCs (cyclic nucleotide-gated calcium channels) and ANNs (annexins) (Zheng et al., 2012; Li et al., 2018a), RNA thermometers, alternative splicing, changes in DNA/chromatin structure (H2A.Z), photoreceptor phytochrome B, and EARLY FLOWERING 3 (ELF3), which exhibits liquid–liquid phase separation. Recent studies have shown that TT3.1 located on the plasma membrane can transfer heat signals to chloroplasts and improve the thermotolerance of rice (Zhang et al., 2022a). Together, these thermosensors sense high temperatures in the environment and efficiently, repeatedly, and stably transmit heat signals to downstream response factors (Vu et al., 2019; Guihur et al., 2022). On the other hand, research into rice has mainly focused on the response to high-temperature stress in recent years, but it is well established that high-temperature stress can cause protein misfolding and accumulation of high levels of reactive oxygen species (ROS) in rice, which reduce plant thermotolerance (Li et al., 2022).

### 3.1 Heat stress and transcriptional regulation

When plants are under heat stress, they initiate a heat shock response, which is induced by the accumulation of denatured proteins and the transient influx of calcium through specific calcium-permeable channels in the plasma membrane (Zheng et al., 2012; Li et al., 2018a). When rice is subjected to heat stress, a series of heat shock genes are induced, among which heat shock proteins (HSPs) and heat shock factors (HSFs) play crucial role in plant thermotolerance. HSPs can be divided into HSP100, HSP90, HSP70, HSP60, HSP40, and small HSPs; these proteins defend cells against damage (Muller and Rieu, 2016; Wang et al., 2020). HSPs function as molecular chaperones to enhance protein folding and prevent the accumulation of unfolded proteins in cells under high-temperature stress (Ding et al., 2020). OsHSP101 positively regulates tolerance to high temperature and acquired heat shock memory in rice. *CHLORPLAST-LOCALIZED HEAT SHOCK PROTEIN 70* (*OsHSP70CP1)* is essential for chloroplast development under high-temperature conditions (Kim and An, 2013). In addition to HSPs, other related components have been discovered and placed in a complex regulatory network. HSFs are key components of the plant heat-stress response that play important roles in heat-stress memory. To date, 25 *OsHSF* genes have been identified in rice (Mittal et al., 2009). Exogenous expression of *OsHSFA2e* enhanced thermotolerance in transgenic *Arabidopsis* (Yokotani et al., 2008). Under high-temperature conditions, *OsHSFA2d* is alternatively spliced into *OsHSFA2dI*, the transcriptionally active form of *OsHSFA2d*, which participates in the heat-stress response (Cheng et al., 2015).

### 3.2 Heat stress and physiological adaptations

High-temperature stress inhibits anthocyanin accumulation in rice by suppressing the expression of various genes in the anthocyanin biosynthetic pathway. On the other hand, anthocyanins are widely recognized as antioxidant factors in plant cells with high ROS scavenging ability. And it is known that high-temperature also causes the accumulation of ROS in plant cells, which eventually leads to plant death. The presence of antioxidants is necessary to maintain the dynamic balance of ROS in plants and protect them from the adverse effects of high-temperature stress (Zaidi et al., 2019). High-temperature stress leads to the production of reactive oxygen species, which in turn disrupts the membrane system of cysts, chloroplasts, and plasma membranes. Inactivation of the photosystem, reduction of photosynthesis and inactivation of rubisco affect the production of photoassimilates and their distribution. This ultimately affects flowering, seed filling, size, number, and maturity of rice seeds, thus hindering crop productivity (Miyazaki et al., 2013; Lal et al., 2022). In addition, high temperature is also one of the environmental factors that cause premature leaf senescence in plants. Respiration is very sensitive to high temperatures. The abundance of CYTOCHROME C OXIDASE (COX), the key protein of respiration, decreased significantly when subjected to prolonged high temperature stress (Rashid et al., 2020). The decrease in respiration rate was predicted to be greater for thermotolerant rice (Ferguson et al., 2020). In order to reduce the damage to the plant, when suffer from high temperatures, the leaves open their stomata by increasing transpiration, which in turn has the effect of reducing the surface temperature of the leaves. On the other hand, sustained high temperatures can lead to plant death by causing massive water loss, reduced membrane mobility and permeability, and stomatal closure (Cochard et al., 2007; Liu et al., 2016).

### 3.3 Heat stress and phytohormones

Plant hormones also regulate thermotolerance in rice *via* independent or interconnected pathways. Moreover, the application of exogenous plant hormones affects the thermotolerance of rice (Fahad et al., 2016). Salicylic acid (SA) enhances stress resistance in plants and plays an important role in regulating rice thermotolerance. Under high-temperature conditions, SA reduces the accumulation of ROS in anthers to prevent premature degradation caused by tapetal programmed cell death, thereby alleviating pollen abortion (Feng et al., 2018; Nadarajah et al., 2021). Brassinosteroids (BRs) function in plant morphogenesis and abiotic stress tolerance. Application of BRs can significantly improve the thermotolerance of plants (Ren et al., 2022). And BRs induce thermotolerance in plants under high-temperature stress by increasing the synthesis of HSPs and the expression of genes encoding protective enzymes (Hwang and Back, 2019). Ethylene-mediated signaling pathways help reduce oxidative damage, maintain chlorophyll content, and enhance thermotolerance in rice seedlings under high-temperature stress. Ethylene also affects rice thermotolerance by regulating carbohydrate metabolism, antioxidant systems, and thus photosynthesis (Gautam et al., 2022).

In general, Abscisic acid (ABA) normally plays a positive regulatory role in rice in response to high-temperature stress. For example, ABA can prevent pollen abortion under high-temperature stress by regulating sugar metabolism in rice spikelets (Rezaul et al., 2019). Rice endogenous ABA content can be regulated by *9-CIS-EPOXYCAROTENOID DIOXYGENASE* (*OsNCED1*), which positively regulates thermotolerance in rice seedlings by increasing endogenous ABA content, thereby improving antioxidant capacity, and activating the expression of heat and ABA-related genes expression (Zhang et al., 2022b). However, ABA also plays a negative regulatory role in regulating thermotolerance in rice. High temperature causes the accumulation of ABA in rice, which inhibits seed germination (Liu et al., 2019). ABA is a negative regulator of thermotolerance in *high temperature susceptibility* (*hts*) plants with semi-rolled leaves by modulating energy homeostasis. More interesting, exogenous ABA significantly decreased thermotolerance of *hts* plants, but clearly enhanced thermoresistance of WT (Li et al., 2020b). The function of ABA on heat stress is important and comprehensive. More research work may be needed to clarify the comprehensive function of ABA in response to heat in detail. Jasmonic acid (JA) and its methyl ester play important roles in plant responses to biotic and abiotic stresses. HEAT-TOLERANCE GENE ON CHROMOSOME 3 (HTG3) regulates thermotolerance in rice by upregulating two heat-responsive *JASMONATE ZIM-DOMAIN* (*JAZ*) genes (Wu et al., 2022a). Auxin plays an important role in maintaining spikelet fertility, and decreased levels of the active auxin indole-3-acetic acid (IAA) can cause pollen abortion. High temperature stress induced rice spikelet sterility by inhibiting pollen tube elongation and interfering with auxin homeostasis in the pistil (Zhang C et al., 2018; Zhang N et al., 2018).

Increases in global temperature seriously affect rice yields, making it crucial to understand the roles of different phytohormones and their crosstalk in regulating the thermotolerance of rice. To date, few studies have focused on the roles of endogenous plant hormone signaling in thermotolerance in rice. This line of research will be crucial for maintaining the growth and yield of rice and enhancing its ability to withstand adverse environmental conditions. Designing strategies to reduce yield losses in rice may involve using the components of phytohormone signaling networks to breed varieties with altered expression levels of important stress-response regulators.

### 3.4 Heat stress involved in complex stresses

A variety of stresses co-occur in rice fields, the most common of which are heat and drought stress. The response of rice to combined stress factors is unique, with rapid adjustments at the physiological and molecular levels (Yadav et al., 2022). High temperatures are often associated with water deficit. Plants normally close their stomata in response to rapid water loss from plant tissues or the soil, but this can lead to increased tissue temperatures due to impaired transpiration. In addition, high temperatures can lead to drought stress through evapotranspiration (Jin et al., 2015). Exposure to combined heat and drought stress can lead to the production of ROS, triggering protective responses such as increasing the expression and activity of ROS-scavenging enzymes and molecules. Rice senses stress signals through signaling sensors including Ca^2+^ and ROS to activate transcription factors such as bZIP, MYB/MYC, WRKY, AP2/EREBP, and NAC (Yang et al., 2022). In addition, increasing the expression of *CATALASE* (*CAT*), *ASCORBATE PEROXIDASE* (*APX*), and *GATA28a* genes helped protect thermotolerant rice variety N22 from lethal damage when heat and drought co-occurred (Yadav et al., 2022). In some parts of the world, where salt stress and high-temperature stress coexist, the accumulation of flavonoids helps regulate endogenous phytohormone levels in rice and balances Na^+^/K^+^ levels, allowing plants to resist combined salt and heat stress (Jan et al., 2021).

Some genes might help rice survive under combined high-temperature and salt stress. For example, overexpression of *SATIVA INTERMEDIATE FILAMENT* (*OsIF*) helped stabilize photosynthetic mechanisms under both stresses, resulting in significantly higher yields than those of the WT (Soda et al., 2018). *DEHYDRATION-RESPONSIVE ELEMENT-BINDING PROTEIN 1C* (*OsDREB1C*) positively regulated heat and salt tolerance (Wang et al., 2022c). Moreover, *DEHYDRATION-RESPONSIVE ELEMENT-BINDING PROTEIN 1C* (*OsDREB1G*) and *SAP AND MIZ 1* (*OsSIZ1*) positively regulated drought, heat, and salt tolerance (Mishra et al., 2018; Wang et al., 2022c).

Most studies of the effects of abiotic stress on rice reported to date have focused on the effects of a single stress on plant growth and development. However, rice often faces stressful conditions in addition to high-temperature stress, making it crucial to study the effects of combinations of stresses. Studying the coping mechanisms of rice under combined stress can help maintain plant growth and improve yields in the face of complex environments.

## 4 Phenotypes associated with high-temperature adaptation in rice

The development of thermotolerant varieties is an important goal of rice improvement. Finding quick and convenient methods for identifying them will help achieve this goal. Diurnal respiration (Rd) is the most plastic physiological characteristic of plants under high-temperature stress. Studies have predicted that plants with higher thermotolerance would show greater decreases in respiration rates under high-temperature stress (Ferguson et al., 2020). However, more research is needed to confirm this notion. Nevertheless, we can preliminarily predict that a certain variety has strong thermotolerance by examining the following thermotolerance-related phenotypes (**Table 1**) in rice and perhaps other plant species.

**Table 1:** Thermophenotypes and physiological phenotypes of rice.

Gene	Expression (loss/over)	Thermophenotype	Organ	Physiological Phenotype
*OsRBG1*	over	thermotolerant	root	Roots and young shoots are longer than WT (Lo et al., 2020).
		(Lo et al., 2020).		
*OsNSUN2*	loss	thermosensitive	root	Roots are significantly shorter at the four-leaf stage (Zhu and Zhang, 2020).
		(Zhu and Zhang, 2020).		
*OsRCc3*	over	thermotolerant	root	Roots are stronger than those of the WT (Li et al., 2018b)
		(Li et al., 2018b)		
*OsZFP350*	over	thermotolerant	root	The root volume and length at seedling stage are significantly higher than those of the WT, and the aboveground biomass are also significantly higher than that of the WT (Kang et al., 2019).
		(Kang et al., 2019).		
*OsPL*	loss	thermotolerant	root, leaf	Roots are longer than those of the WT, and the leaves were purple and grew slowly at the later stage of filling (Akhter et al., 2019).
		(Akhter et al., 2019)		
*OsHYR*	over	thermotolerant	root, leaf	The roots are longer and thicker. The leaves are bright and dark green (Ambavaram et al., 2014).
		(Ambavaram et al., 2014).		
*OsPSL50*	loss	thermosensitive	Stem, leaf	The plants are short and prematurely senescent at heading stage (He et al., 2021).
		(He et al., 2021)		
*OsGSK1*	loss	Thermotolerant	stem	The plants are dwarf, short (Koh et al., 2007).
		(Koh et al., 2007).		
*OsPDT1*	loss	thermosensitive	stem	The plants are dwarfed (Deng et al., 2020).
		(Deng et al., 2020).		
*OsSPL7*	loss	thermosensitive	stem	The plants grow slowly and the plants are stunted (Hoang et al., 2019).
*OsFKBP20-1b*	loss	Thermosensitive	stem	The plants have retarded growth (Park et al., 2020).
		(Park et al., 2020)		
*OsHSA1*	loss	thermosensitive	stem, leaf	The plants are short and the leaves are albino. Chloroplast development is slower than WT and eventually returned to green, but the spike is still lighter than those of the WT. (Qiu et al., 2018).
		(Qiu et al., 2018).		
*OsHTS1*	loss	Thermosensitive	stem, leaf	The plants are short, with reduced chlorophyll contents and lighter-colored leaves (Chen et al., 2021a).
		(Chen et al., 2021a).		
*OsHSP40*	loss	Thermosensitive	leaf	Leaves are smaller than those of the WT (Wang et al., 2022b).
		(Wang et al., 2022b)		
*OsMDHAR4*	over	Thermosensitive	leaf	The plants have lower percentages of completely closed stomata than WT after high temperature treatment (Liu et al., 2018),
		(Liu et al., 2018)		
*OsqEMF3*	near-isogenic line IR64 + *qEMF3*	thermotolerant	flower	The plants advance flowering by about 2 hours (Bheemanahalli et al., 2017).
		(Bheemanahalli et al., 2017)		

### 4.1 Root phenotypes at high temperature

Roots play a vital role in absorbing, transporting, and storing water and nutrients, as well as anchoring plants. Thermotolerant rice varieties usually have larger (including more lateral roots and branches) and stronger root systems compared with thermosensitive varieties (Tiwari et al., 2022). For example, plants overexpressing *Big Grain 1* (Os*RBG1*) showed enhanced thermotolerance, and three-week-old plants had longer roots than the WT (Lo et al., 2020). Plants overexpressing *HIGHER YIELD RICE* (*OsHYR*) also had more adventitious roots, with greater length and thickness, than the WT (Ambavaram et al., 2014). *ZINC-FINGER PROTEIN* (*OsZFP350*)-overexpressing plants showed enhanced thermotolerance, and root volume and length at the seedling stage were significantly larger than those of the WT under normal conditions (Kang et al., 2019), as was the case for *OsRCc3* (*encoding a rice root special gene*)-overexpressing plants (Li et al., 2018b). Loss-of-function mutants of *PURPLE LEAF*(*OsPL*) showed enhanced thermotolerance and had longer roots than the WT (Akhter et al., 2019). By contrast, loss-of-function mutants of *OsNSUN2* (encoding an RNA 5-methycytosine methyltransferase) were thermosensitive and had significantly shorter roots than the WT (Tang et al., 2020; Zhu and Zhang, 2020). Therefore, examining root length, number of lateral roots and branches, and root biomass can provide important information for studying plant thermotolerance.

### 4.2 Stem phenotypes at high temperature

Plant height reflects the growth state of a plant and may therefore be related to thermotolerance. Loss-of-function mutants of *OsHST1* (encoding a β-ketoacyl carrier protein reductase) showed severe hypersensitivity to high temperature at the seedling stage and a dwarf phenotype under normal conditions (Rana et al., 2019; Chen et al., 2021a). Similarly, loss-of-function mutants of *PHOTOPERIOD-THERMOSENSITIVE DWARFISM 1* (*OsPTD1*), *SPTTED LEAF 7* (*OsSPL7*), *HEAT-SENSITIVE ALBINO 1* (*OsHSA1*), and *OsFKBP20-1b* (belonging to the immunophilin family) showed decreased thermotolerance and a stunted phenotype under normal conditions (Qiu et al., 2018; Hoang et al., 2019; Deng et al., 2020; Park et al., 2020). By contrast, *OsZFP350*-overexpressing plants were more thermotolerant and taller than the WT under normal conditions (Kang et al., 2019). Therefore, rice thermotolerance may be related to plant height, that is, thermosensitive plants are short, and thermotolerant ones are tall. However, not all plants conform to this rule. For example, loss-of-function mutants of *GLYCOGEN SYNTHASE KINASE 3-LIKE GENE 1* (*OsGSK1*) showed decreased thermotolerance, but plants overexpressing *OsGSK1* showed a dwarf phenotype (Koh et al., 2007). Further experiments are required to examine the potential connection between plant height and thermotolerance.

### 4.3 Leaf phenotypes at high temperature

As the main sites of photosynthesis and transpiration and the largest organs exposed to the environment, leaves play an important role in plant growth and development. Leaf size, color, shape, and other morphological characteristics directly affect photosynthesis in rice, thus affecting grain yield (Kumar et al., 2021; Vitoriano and Calixto, 2021). Leaves of loss-of-function mutants of *OsHSP40* were significantly smaller than those of the WT (Barghetti et al., 2017; Wang et al., 2022b). Leaf color is an important morphological characteristic in rice breeding (Liu et al., 2012). Chlorophyll content of the thermosensitive mutant of *OsHTS1* (encoding a thylakoid membrane-localized β-ketoacyl carrier protein reductase) was lower than that of the WT, and leaf color was lighter (Chen et al., 2021a). Chloroplasts of the thermosensitive mutant of *OsHSA1* developed slowly and showed albinism, which was alleviated during growth, but leaf color was still lighter in the mutant than in the WT (Qiu et al., 2018). By contrast, thermotolerant *OsHYR*-overexpressing plants had darker green leaves than the WT (Ambavaram et al., 2014).

We propose that leaf color is related to thermotolerance in rice; thermotolerant rice generally has dark leaves, and thermosensitive rice has lighter leaves. Bread wheat genotypes of the stay-green type showed high chlorophyll content and low canopy temperature under both control and high-temperature stress conditions. Maintaining the green phenotype can mitigate the harmful effects of high-temperature stress by maintaining grain yield and biological production (Latif et al., 2020). Increasing chlorophyll content also improved thermotolerance in ryegrass. Treating ryegrass with sodium copper chlorophyllin increased chlorophyll accumulation, downregulated chlorophyll catabolic and senescence genes, and enhanced H_2_O_2_ scavenging by the peroxidase pathway, thus inhibiting heat-induced leaf senescence (Yu et al., 2022). Therefore, measuring the chlorophyll content and photosynthetic rate of plants may help in screening plants for thermotolerance. In addition, the senescence rate of leaves, stomatal opening, and leaf temperature regulation might be used as an indicator to identify the thermotolerance of rice. *PREMATURE SENESCENCE LEAF 50* (*PSL50*) mutant are highly sensitive to high temperatures (He et al., 2021). Inhibition of *MONODEHYDROASCORBATE REDUCTASE* (*OsMDHAR4*) expression enhances thermotolerance by inducing stomatal closure in rice plants (Liu et al., 2018). Increasing leaf temperature negatively regulates the thermotolerance of *hts* mutant (Li et al., 2020b). Leaf area and thickness could also be used as an index to identify thermotolerant plants. Thicker leaves have higher chlorophyll content and photosynthetic efficiency. Moreover, the larger the leaf area, the higher the relative number of stomata and the transpiration rate and thus the better the heat dissipation and the thermotolerance.

### 4.4 Flowers phenotypes at high temperature

Abnormalities in both anther and ovary development affect spikelet fertility. The well-known highly thermotolerant rice variety N22 showed higher spikelet fertility than the WT after high-temperature stress (Poli et al., 2013; Shi et al., 2022). Altering the time of flowering, such as flowering early in the morning, could help protect crops from high-temperature stress in the environment, thus reducing the damage that crops suffer during flowering. The near-isogenic line IR64 + *qEMF3* advanced flowering time by about 2h, overcoming heat-induced spikelet sterility (Bheemanahalli et al., 2017). Moreover, *qEMF3* advanced the flowering time of Nanjing 11 by about 1.5 h, thereby improving the thermoresistance of this variety (Hirabayashi et al., 2015; Jagadish, 2020). Therefore, statistical analysis of flowering time and spikelet fertility might represent another effective means to identify thermoresistant rice varieties.

## 5 Concluding remarks

With the gradual increase in the global population, the worldwide demand for crops is increasing. By 2050, the global agricultural output may need to increase by 60%–110% to meet the demands of the growing population (Ray et al., 2013). However, over the past century, global temperatures have been steadily increasing, which poses great challenges to the global economy and food security. The rise in ambient temperature is a complex variable with many effects depending on duration and degree. Different plant species have relatively stable optimum growth temperatures throughout their life cycles, and the same plant also has different optimum growth temperatures at different stages of growth and development. Hence, plants have evolved complex, rigorous mechanisms to regulate growth and development to adapt to changing environmental temperatures. When the ambient temperature exceeds the optimum temperature for a plant, the plant is in a state of high-temperature stress.

In view of the complexity of plant responses to high temperature and the importance of rice for global agriculture, this review provides an important theoretical basis for identifying new thermotolerant rice varieties according to the physiological morphology and molecular regulatory mechanisms involved in plant growth and development under high-temperature conditions. Higher than optimum temperatures during each period of rice growth and development affect the overall plant growth. High temperatures can lead to delayed germination, reduced pollen viability, and abnormal ovary development and affect the accumulation of starch grains, among other phenotypes, thus affecting rice production (Chen et al., 2017; Zhao et al., 2018; Afzal et al., 2019; Liu et al., 2019).

In recent years, many studies have focused on how to mitigate the effects of high-temperature stress in rice, but we still have a long way to go before fully understanding the thermotolerance mechanism of rice, optimize its quality, and improve its ability to cope with high-temperature stress. The roles of HSPs and HSFs in high-temperature stress are still an important focus of research on rice thermotolerance (Afzal et al., 2019; Wang et al., 2022b). The high-temperature stress response mechanisms in the model organism *Arabidopsis* have been well investigated by previous studies, the findings of which can be leveraged to expand our understanding of thermotolerance in rice.

This paper also summarizes other physiological phenotypes in roots, stems, leaves, and flowers thereby providing a theoretical basis for further research on genetic variation of rice and screening of thermotolerant varieties. We suggest that root length, leaf color, plant height, flowering time, spikelet fertility and grain filling should be studied as preliminary and rapid statistical indexes for thermoresistant varieties. In addition, leaf senescence rate, stomatal opening and leaf temperature are also very important for rice to cope with high temperature stress. This research direction can help to rapidly screen thermotolerant rice varieties and improve the resistance of rice to unfavorable environmental conditions, but little research has been conducted on this aspect. Of course, the thermoresistant varieties selected need to be moved from the natural living environment into the laboratory to conduct in-depth exploration of their endogenous regulation and metabolic ability, which will be a future research direction. And effects of high temperature on grain quality are also meaningful research filed in the future. The above provides a theoretical basis for ensuring sustainable agricultural development in the face of global warming, as well as achieving high, stable, and safe crop yields to meet the demand for food production from a growing global population.

## Author contributions

All authors listed have made a substantial, direct, and intellectual contribution to the work and approved it for publication.
